# Hyperbolic discounting underpins response curves of mammalian avoidance behaviour

**DOI:** 10.1098/rsbl.2024.0054

**Published:** 2024-07-24

**Authors:** Michael A. Patten, Jutta C. Burger

**Affiliations:** ^1^ Faculty of Biosciences and Aquaculture, Ecology Research Group, Nord University, Steinkjer, Trøndelag, Norway; ^2^ California Invasive Plant Council, Berkeley, CA, USA

**Keywords:** anthropogenic disturbance, behavioural avoidance, human density, response curve

## Abstract

As humans clear natural habitat, they are brought into increased conflict with wild animals. Some conflict is direct (e.g. elevated exposure of people to predators), some indirect (e.g. abandoning suitable habitat because of human activity). The magnitude of avoidance is expected to track frequency of human activity, but the type of response is an open question. We postulated that animals do not respond passively to increased disturbance nor does response follow a power law; instead, their ability to estimate magnitude leads to ‘discounting’ behaviour, as in classic time-to-reward economic models in which individuals discount larger value (or risk) in more distant time. We used a 10-year camera dataset from southern California to characterize response curves of seven mammal species. Bayesian regressions of two non-discounting models (exponential and inverse polynomial) and two discounting models (hyperbolic and harmonic) revealed that the latter better fit response curves. The Arps equation, from petroleum extraction modelling, was used to estimate a discount exponent, a taxon-specific ‘sensitivity’ to humans, yielding a general model across species. Although discounting can mean mammal activity recovers rapidly after disturbance, increased recreational pressure on reserves limits recovery potential, highlighting a need to strike a balance between animal conservation and human use.

## 1. Introduction

A key aspect of an animal’s cognitive assessment of habitat suitability is the possibility of *avoidance*, an assessment that has its own consequences [1–4]. Theory, generally couched in the form of non-consumptive predation, has focussed on disturbance frequency, resource distribution and exposure risk [5–7]. Many of these ideas have been brought under the umbrella of the ‘landscape of fear’, a term that dates to animal response to reintroduced predators [8] but subsequently gained traction to refer to any spatial variation in perceived risk of predation and how this variation affects trophic dynamics and ecosystem function [9,10].

Animal avoidance of human presence, with attendant shifts in diel activity or space use, is increasingly well established [11–15], both for overall abundance and species richness [16]. An outstanding question remains: to what extent does disturbance intensity matter? Theory predicts that ‘fear’ intensity will affect response in a nonlinear manner [1] and that perceived risk asymptotes as disturbance intensity increases [17], even if an inflection point is unknown because response to disturbance varies among species [12,18]. Empirical evidence is less clear: findings are heterogeneous, with no clear pattern of effects of higher human traffic or tendency to habituate [19], perhaps because expected responses of animals to human presence are complex and potentially contradictory, with some species avoiding people while others become commensal [20]. It, nevertheless, has become clearer that human presence and human density affect animals differently [21], and some studies have reported a relationship between increased avoidance and increased vehicular traffic [22,23] or human activity [24].

Animal response to predator presence is well established [1], and humans often elicit the same response as a predator even if their presence is benign [2]. The challenge is to characterize more explicitly organismal response to humans. Is avoidance driven by the *presence* of humans or by the *frequency* of humans? If the intensity of human use matters, at what level (frequency) does it most matter? Most important, what is the theoretical form of the relationship between intensity and animal occurrence? Different forms of a declining curve imply different mechanisms that affect avoidance response—each makes a different assumption about animal perception of threats. We consider four models of curvilinear decay, two of which are prevalent in ecology and two of which have seldom been considered in ecology. The prevalent models are those of exponential decline and inverse polynomial following a basic power law. Exponential decline assumes that perceived risk is a constant function of disturbance intensity: for each increase in mean frequency of humans, there will be a direct decrease in occurrence (increase in avoidance). Power laws—one metric varying by the exponent of another metric—are widespread in natural systems [25] and likewise assume constant perceived risk. The other two models we considered incorporate inconstant perception and are grounded in the concept of ‘discounting’.

Discounting has its roots in behavioural economics and associated psychology of impulsivity. In technical terms, such a model includes a time-to-reward adjustment, a subjective devaluing of a reward as time elapsed until payout increases; i.e. a subject ‘discounts’ (i.e. values less) future pay-offs relative to more immediate pay-offs even when immediate pay-offs are less [26]. The paradigm has shaped thinking on impulsive behaviours (drug use and gambling) and financial decisions and is nearly always applied to temporal questions. Here, we substitute disturbance intensity for time; that is, rather than consider how an animal might maximize its reward rate per unit time [27], we focus on how an animal might minimize its perceived risk per unit disturbance. Discounting is a function of perceived risk, a notion that can apply to our daily lives. Should I drive to the market or walk there? Taking the car is quicker and less wearying physically, yet driving contributes to global heating and lack of exercise carries costs, both long-term concerns. Often the decision to drive discounts future risk for short-term gain. Our two discounting models differ only in how discounting was integrated. The hyperbolic decay model assumes that response is a variable function of disturbance, with avoidance declines at a gradually slower rate as the amount of disturbance increases. The related harmonic decay (also known as ‘rational’) model assumes the decline rate of change (discount) is constant across disturbance intensities. Each of these models (and the exponential model, too) is a special case of the Arps equation [28] from petroleum extraction engineering, use of which allowed us both to identify species-specific responses and to understand the shape of the response curves.

We used extensive data from a camera-trap array to assess which theoretical model best fit response curves, that is to determine how risk perception is related to disturbance intensity. We posited that mammals discounted perceived risk; hence, we predicted that an exponential model would drop too steeply with the lowest levels of disturbance whereas a power law model would not drop steeply enough. When we found support for a discounting model, we used the Arps equation to estimate the exponent, knowledge of which helped us to distinguish between hyperbolic (variable discount) and harmonic (constant discount) models. Finally, we explored consequences of response to disturbance via both an exploration of resilience to ‘bursts’ of human activity and changes over time in mean disturbance intensity. Ultimately, if a discounting model best accounts for the data then it implies that animals respond to humans (and attendant disturbance) in a non-constant manner, which could inform management of reserves, balancing of nature and tourism, and design of mitigation and monitoring plans.

## 2. Methods

Over 50 camera traps (Cuddeback Expert 3300 or HCO Scoutguard SG-565F) were placed in a complex of urban-adjacent parks with restricted public access in central and coastal Orange County, California [12,14]. Cameras were placed inconspicuously (to minimize either spooking animals or drawing unwanted human attention) along trails (62%), at watering troughs (12%) or in vegetation away from either putative ‘concentration point’ (26%). Herein we analyse same-day data collected by 50 cameras June 2007–2016. We defined a ‘day’ as human activity <24 h prior to mammal detection [14]. We pooled data across cameras because our goal was akin to the goal of a meta-analysis: we wished to obtain an average estimate for a species across the study area (to minimize spatial variability). Not all cameras operated continuously over that period, although 28 of them recorded data continuously from June 2007. To avoid overcounting people, human or human-related detection at a given camera within 1 h was summed to a single record, whereas any mammal species detected repeatedly within a 5 min period was tallied as a single record, which yielded detection rates near *p* = 1 [14]. In the end, our extensive dataset included >1 00 000 identifiable mammal records across a full decade of passive sampling.

Each of the four models, we considered has a single predictor, *x*, disturbance intensity, a count of ‘people’ in the broad sense that could include individual hikers, vehicles, cyclists, etc., two taxon-specific parameters that determine the response curve’s shape, *y*_0_, prevalence in the system (effectively a *y*-intercept) and *ς*, a species’ ‘sensitivity’ to disturbance (a measure of the level of risk a species perceives). Two models without discounting were exponential decay,


$$y=y_{0}e^{-\varsigma x},$$

which assumes a constant response rate—for each additional person, there will be a direct decrease in occurrence (increase in avoidance)—and an inverse polynomial, which can take many forms. We modelled a basic inverse-square form


$$y=y_{0}+\frac{\varsigma }{x^{2}},$$

for consistency with a wide variety of power-law formulations. Two models with discounting were hyperbolic decay of the form


$$y=\frac{y_{0}\;\varsigma }{\varsigma +x}\;,$$

which assumes that response is discounted proportionally to disturbance and harmonic decay (also known as ‘rational’) of the form


$$y=\frac{y_{0}}{1+\varsigma x},$$

which assumes the decline rate of change (discount) is constant across disturbance. The hyperbolic, harmonic and exponential models are special cases of the Arps equation [28]:


$$y=\frac{y_{0}}{(1+d\;\varsigma x{)}^{1/d}},$$

where *d* is the discount, the change in the ‘loss ratio’ *y*/Δ*y* [28], hence *d* = Δ(*y*/Δ*y*). When *d* = 0 (i.e. no discount), then the equation, at its limit, reduces to the exponential model. When *d* = 1 (i.e. constant discount), then the equation reduces to the harmonic model. When 0 < *d* < 1 (i.e. inconstant discount, proportional to *x*), then the equation is one form of a hyperbolic model.

We fit models for the seven species photo captured most frequently: *Lynx rufus* (bobcat), *Puma concolor* (puma or mountain lion), *Urocyon cinereoargenteus* (grey fox), *Canis latrans* (coyote), *Mephitis mephitis* (striped skunk), *Procyon lotor* (northern racoon) and *Odocoileus hemionus* (mule deer) and for all species combined. The response variable was the proportion of taxon-specific captures for days with 0, 1, 2, … humans counted. Each of the four theoretical models was fit as a Bayesian regression built with customized JAGS code and run via the ‘rjags’ package in R [29]. All priors for estimated parameters were flat, with a uniform distribution. We assessed relative model fit by means of the Watanbe–Akaike information criterion (WAIC; also known as the ‘widely applicable information criterion’), which is fully Bayesian in that it is obtained from the posterior distribution rather than from a point estimate [30].

We quantified resilience using mammal captures 3 days in advance of and 2 days following ‘wilderness access days’, during which areas either normally closed or of otherwise limited access are opened to the public. These days spark a pulse of people, typically of the order of a 10-fold increase in disturbance. In this case, our interest was an estimate of activity, *A*, a metric of relative (not absolute) abundance, at *t* − 3, *t* − 2, … *t* + 2 days around an event on day *t*. We estimated *A* for each of the *j* days (*j* = 6) with a Bayesian negative binomial model,


$$y_{j}∼\;negative\;binomial(\pi_{j},\;r_{j})$$

$$\pi_{j}=r_{j}\;/\;(r_{j}-\mu_{j})$$

$$A_{j}=r_{j}(1-\pi )/\pi_{j}$$

with flat gamma priors for the mean, *μ*, and a gamma prior for overdispersion, *r*, with the rate parameter flat but the shape parameter set to the mean number of mammal detections over the 6 days. For this model, *y* was the number of mammal detections at seven cameras in Limestone Canyon, where wilderness access days took place.

Our final analysis was to use quarterly data (i.e. every three months) across the whole of study period (*n* = 37 quarters) in a time-series analysis of trends in mammal detections and human disturbance, the goal being to infer whether, say, mammal detections decreased over time while controlling for seasonality or other lags. The time-series analysis was run via ‘proc arima’ in SAS Statistical Software.

## 3. Results

**Figure 1.**
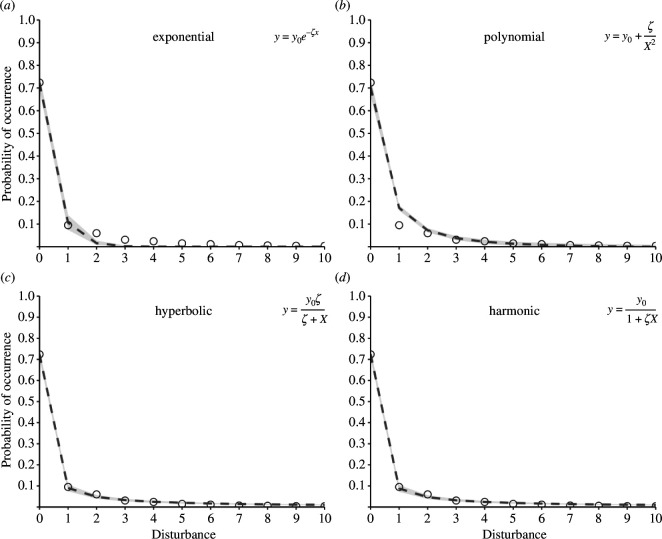
Fit (regression line ± 95% highest density credible intervals) for probability of photo capture across all mammals for (*a*) exponential, (*b*) inverse polynomial, (*c*) hyperbolic decay and (*d*) harmonic decay models. Points are the proportion of camera-trap ‘captures’ for a given number of humans. Exponential models invariably underestimated response at higher levels of disturbance for each of the seven study species. In contrast, inverse polynomial models usually underestimated response at low levels of disturbance. Hyperbolic and harmonic models often yielded similar fit, with, in general, hyperbolic models tending to be slightly better.

Exponential models never fit response curves well, and only for one species did an inverse polynomial model best fit the data (figure 1, table 1); instead, discount models fit response curves much better (figure 1), with hyperbolic decay models performing slightly better than harmonic decay models (table 1). Estimates of the discounting rate, *d*, support a hyperbolic decay model with *d* = 0.64−0.86, values generally near to the *d* = 1 for harmonic decay given that for most taxa the upper 95% highest density credible interval was ≈ 1 (table 2).

**Table 1.** Model fit of the four basic models and from the Arps (1945) equation, which fits a hyperbolic model with discounting, *d*, allowed to vary 0 < *d* < 1. The ‘best’ (lowest WAIC) of the four basic models is italicized; the ‘best’ model overall has bolded text. Hyperbolic decay models nearly always provided the best fit, although fit differed little for harmonic decay models. Hyperbolic models with *d* estimated from the Arps equation [28] generally were better still.

model	combined	*Lynx rufus*	*Puma concolor*	*Urocyon cinereoargenteus*	*Canis latrans*	*Mephitis mephitis*	*Procyon lotor*	*Odocoileus hemionus*
exponential	−112.15	−98.73	−102.63	−110.54	−95.67	−106.68	−134.96	−125.43
inverse polynomial	−96.90	−128.53	−98.96	−107.15	−123.11	**−134.05**	−69.52	−83.71
hyperbolic decay	*−142.78*	**−141.91**	*−133.50*	*−136.39*	*−133.58*	−121.32	−148.09	*−143.05*
harmonic decay	−142.71	−141.83	−133.41	−136.07	−133.40	−121.11	**−148.37**	−142.85
Arps equation	**−145.25**	−141.89	**−136.02**	**−143.09**	**−137.84**	−132.93	−147.86	**−146.46**

**Table 2.** Parameter estimates [±95% highest density credible intervals] from the Arps equation [28] for a hyperbolic model. Parameters are prevalence in the system when disturbance is 0 (*y*_0_; effectively an intercept), a taxon-specific ‘sensitivity’ (*ς*; higher is more sensitive) and the discounting metric (*d*). Note that estimates of *d* vary less than the other parameters and tend towards a harmonic model (*d* = 1) and well away from an exponential model (*d* = 0), a signal that discounting is a feature of mammalian response to human disturbance.

species	*y*_0_	*ς*	*d*
*Lynx rufus*	0.594 [0.572, 0.615]	3.50 [2.50, 4.50]	0.859 [0.699, 1.000]
*Puma concolor*	0.720 [0.698, 0.741]	4.57 [2.95, 6.38]	0.780 [0.591, 0.999]
*Urocyon cinereoargenteus*	0.711 [0.690, 0.732]	3.88 [2.58, 5.48]	0.706 [0.494, 0.947]
*Canis latrans*	0.505 [0.484, 0.528]	2.03 [1.51, 2.60]	0.764 [0.581, 0.961]
*Mephitis mephitis*	0.616 [0.592, 0.638]	2.49 [1.76, 3.32]	0.639 [0.419, 0.868]
*Procyon lotor*	0.847 [0.826, 0.868]	12.48 [5.06, 20.52]	0.823 [0.558, 1.000]
*Odocoileus hemionus*	0.798 [0.777, 0.819]	6.24 [3.35, 9.72]	0.733 [0.494, 0.997]
combined	0.724 [0.703, 0.745]	5.00 [3.08, 7.08]	0.784 [0.586, 1.000]

**Figure 2.**
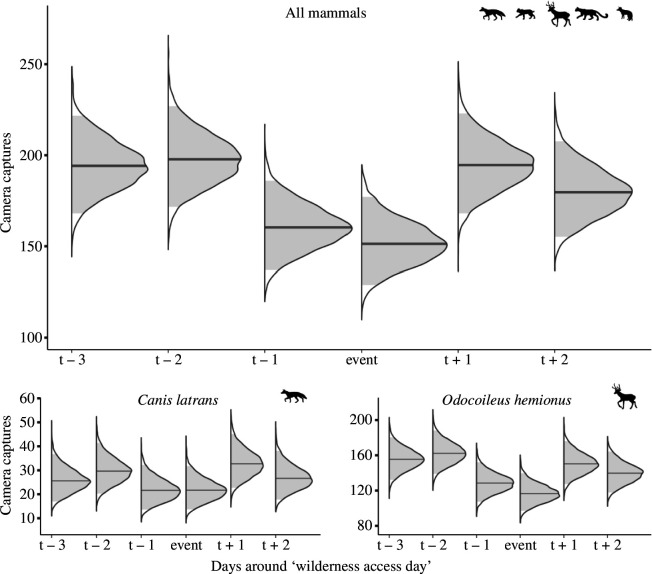
Camera trap detections per day before and after ‘wilderness access days’ in Limestone Canyon, California, during which access restrictions were eased, spurring a 10-fold increase in human disturbance. Results are posterior distributions of image captures (*n*) from a Bayesian negative binomial model and are presented for five mammal species combined and for the two species recorded most often, *Canis latrans* (coyote) and *Odocoileus hemionus* (mule deer).

**Figure 3.**
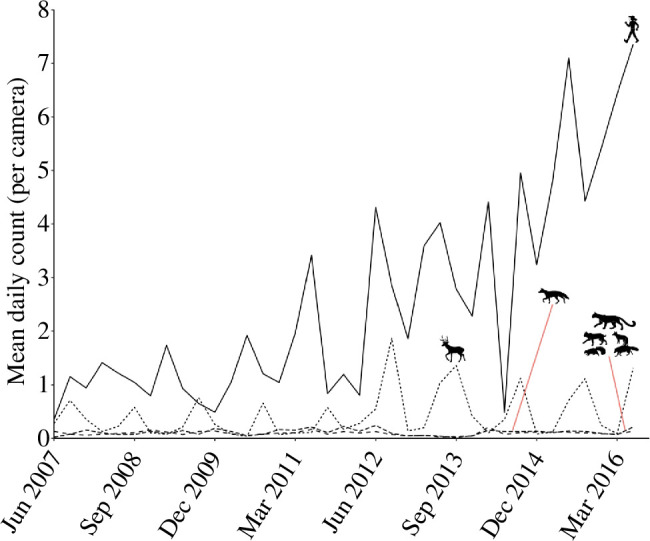
Incidence of humans and mammals, as mean per quarterly sample, across the study area in Orange County, California, from June 2007 to June 2016. Human use of the reserves has increased markedly, whereas mammal detections varied seasonally but otherwise exhibit no trends. Separate lines are shown for *Odocoileus hemionus* (mule deer), *Canis latrans* (coyote) and an aggregation of five species—*Puma concolor* (puma), *Lynx rufus* (bobcat), *Urocyon cinereoargenteus* (grey fox), *Mephitis mephitis* (striped skunk) and *Procyon lotor* (northern racoon).

Mammal encounters were depressed during ‘wilderness access days’ when human presence was markedly higher, yet the effect began a day early and encounters returned to pre-event levels a day later (figure 2). Although wilderness access days caused a spike in disturbance, human use increased sharply over the decade (*χ*^2^_6_ = 52.2, *p* < 0.001), whereas mammal activity held steady once we accounted for seasonality (figure 3; *χ*^2^_6_ = 6.2, *p* > 0.40).

## 4. Discussion

We found strong support for hyperbolic decay response curves, which indicates that mammals discount additional human disturbance proportionally as disturbance increases. It is the first push of disturbance, between one and three people, that triggers the sharpest avoidance response, a response that is both nonlinear and inconstant. The change in response decreases thereafter, such that the difference between five and 10 people is low but detectable yet the difference between 100 and 200 people is negligible. Animals can estimate magnitude [31] and also are responsive to disturbance frequency. An increase of one visitor to two visitors per day to a reserve means, on average, disturbance once per 24 h to once per 12 h, assuming visitors are independent. The difference between 20 and 21 independent visitors per day changes mean frequency of human encounter from 1/72 to 1/68.6 min, a much smaller difference. Whether animals respond to the number of people or the frequency of encounter with them (or both) require further study.

It has been suggested that species-specific responses are rife, impeding efforts to build general models or fashion a general understanding of the effects of human disturbance [32,33]. Our results corroborate taxon-specific response curves, yet our modelling approach accounts for differences via a taxon-specific sensitivity (*ς*), effectively a ‘slope’. Sensitivity can be viewed as a taxon-specific trait associated with, for example, flight initiation distance [18], stress response [34] or degree of acclimation [35]. Hence, *ς* is a measure of a taxon’s *direct* response to human presence and not an indication of its ability or propensity to form commensal relationships with humans or to occupy human-modified habitats. Importantly, this generality means that predictions for a naive system could be generated from an Arps-type model with *d* ≈ 0.78 while allowing *ς* to vary across taxa. It would be worthwhile to estimate *ς* across study areas to assess the extent to which sensitivity varies geographically.

Our data suggest that depressed activity or spatial shifts are most prevalent at low levels of disturbance, generally just one to three or four people. Camera traps tend to underestimate raw counts of humans because of built-in shutter delays and the typically clustered nature of human activity, but underestimates are unlikely to be extreme. Even if off by half, the presence of only a few people is enough to depress mammal encounters, as seen in other systems, as when even low numbers of skiers negatively affected occurrence probability of *Rangifer tarandrus*, the caribou or reindeer [24]. We nevertheless hypothesize that a considerable (but unknown) extent of avoidance behaviour is facultative. A 10-fold increase in human disturbance depressed mammal activity. The specific cause, whether number of people, associated noise, or expanded footprint of human use, is unclear, yet mammal activity rebounded as soon as disturbance returned to pre-disturbance levels. Behavioural responses to lessened disturbance tend to be swift, as with the various documented shifts in bird song, diel activity and occupancy in response to COVID-19 lockdowns [36–38].

We are encouraged by apparent resilience of the system, yet our encouragement is tempered by evidence of a sharp increase in human use of the reserves. If disturbance intensity continues to climb, the study area may transition from a pulse experiment to a press experiment [39], after which, discounting and facultative resilience aside, mammal activity cannot recover. In other words, although mammal species may shift peak activity temporally or spatially to avoid people [11,13], at some point, there is nowhere to shift to or shifting itself brings consequences [14]. Reserve managers already make tough decisions to balance environmental protection and tourism demand. An ability to predict species-specific responses to increased disturbance can lead to management plans wherein, for example, access to certain areas is restricted if it benefits a certain sensitive species, especially if we have a clear idea of how much disturbance is too much.

## Data Availability

Analysed data are available on Dryad [40].
